# Gas-free immersion system with dual-channel scope for rapid hemostasis during gastric endoscopic submucosal dissection

**DOI:** 10.1055/a-2376-1851

**Published:** 2024-08-13

**Authors:** Tatsuma Nomura, Takanobu Mitani, Junki Toyoda, Yuto Ikadai, Tomohiro Sase, Tomonori Saito, Katsumi Mukai

**Affiliations:** 1 Department of Gastroenterology, Suzuka General Hospital, Suzuka, Japan; 2 Department of Gastroenterology, Ise Red Cross Hospital, Ise, Japan

The usefulness of saline immersion for endoscopic submucosal dissection (ESD) has been reported 1 2 . However, hemostasis is difficult to achieve during ESD performed under saline-immersed conditions. Therefore, we have previously reported a gas-free immersion (GFI) dissection technique that can achieve compression hemostasis on the side of a tapered hood without a hole while producing turbulence within the hood 3 4 . In this report, we introduce a GFI system with a dual-channel scope (GIF-2TQ260M; Olympus) for rapid hemostasis during gastric ESD.

In the GFI system, only saline solution is supplied instead of carbon dioxide (CO _2_ ) gas when the gas/water bulb button is pressed ( Fig. 1 , Video 1 ). The GFI system uses a pressure infusion bag, extension tube, and saline solution. First, an extension tube is connected to the water supply connector, so that only water can be delivered. Next, pressure (≥300 mmHg) is applied to the pressure infusion bag. Using this technique, the ESD is performed using hemostatic forceps pre-inserted into one channel. Thus, bleeding occurring during the procedure can be stopped immediately using the hemostatic forceps.

**Fig. 1:**
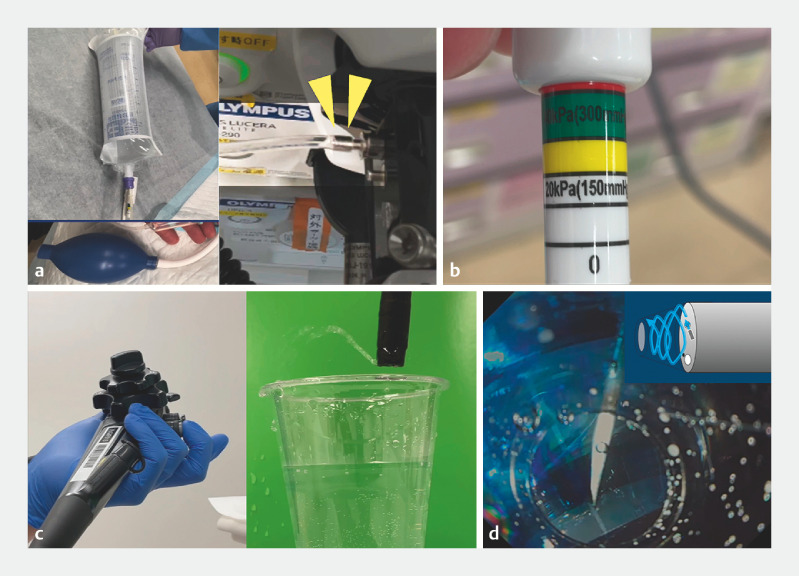
Gas-free immersion system. **a** Pressure infusion bag, saline solution, and extension tubing. The extension tube is connected to the connection port (yellow arrowheads). **b** Pressure (≥300 mmHg) is applied to the pressure infusion bag. **c** Only saline solution is pumped toward the endoscope lens – no carbon dioxide gas or air. **d** Pressing the button creates turbulence in the tapered hood. This prevents bleeding from entering the hood, thus maintaining a good endoscopic view.

Gastric endoscopic submucosal dissection using a gas-free immersion system with a dual-channel scope for rapid hemostasis.Video 1

Our patient was a 78-year-old man with an early-stage tumor in the stomach measuring 30 mm ( Fig. 2 ). We performed an ESD resection under the GFI system with a dual-channel scope. When bleeding occurred, we immediately applied compression using the side of the calibrated, small-caliber-tip, transparent hood while pressing the water bulb button. Hemostasis was then achieved using the coagulation mode with hemostatic forceps. The bleeding point was easily identified because the turbulent flow in the GFI pushed the bleeding out of the hood. The tumor was resected en bloc without adverse events. The defect was completely closed using the reopenable clip-over-line technique 5 .

**Fig. 2:**
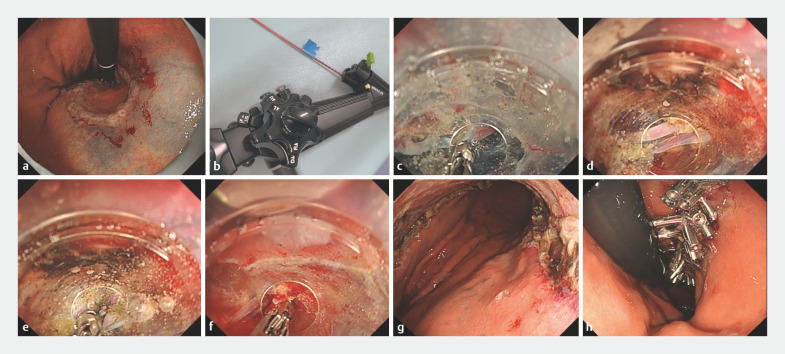
Gastric endoscopic submucosal dissection (ESD) using a gas-free immersion system with dual-channel scope for rapid hemostasis. **a** Endoscopic image after marking and submucosal injection. **b** Hemostatic forceps inserted in advance into an accessory channel. **c** When bleeding occurs, compression hemostasis is performed with the side of the wide calibrated, small-caliber-tip, transparent hood. The hemostatic forceps can be used to apply a coagulation tourniquet immediately. **d, e** If a pulsating artery is detected, the hemostatic forceps can be immediately inserted for precoagulation. **f** The source of bleeding during a mucosal incision can be detected by pressing the button to irrigate the hood with saline solution. **g** Mucosal defect after complete tumor resection by ESD. **h** Mucosal defect completely closed by the reopenable clip-over-line technique. Pathological findings revealed a 30-mm intramucosal adenocarcinoma with negative horizontal and vertical margins.

Under the GFI system, hemostatic forceps can be inserted into one channel of a dual-channel endoscope to stop bleeding immediately during ESD.

Endoscopy_UCTN_Code_TTT_1AO_2AG_3AD

## Notes

Endoscopy E-Videos https://eref.thieme.de/e-videos
*E-Videos* is an open access online section of the journal *Endoscopy* , reporting on interesting cases and new techniques in gastroenterological endoscopy. All papers include a high-quality video and are published with a Creative Commons CC-BY license. Endoscopy E-Videos qualify for HINARI discounts and waivers and eligibility is automatically checked during the submission process. We grant 100% waivers to articles whose corresponding authors are based in Group A countries and 50% waivers to those who are based in Group B countries as classified by Research4Life (see: https://www.research4life.org/access/eligibility/ ). This section has its own submission website at https://mc.manuscriptcentral.com/e-videos .
